# Homeobox regulator Wilms Tumour 1 is displaced by androgen receptor at cis-regulatory elements in the endometrium of PCOS patients

**DOI:** 10.3389/fendo.2024.1368494

**Published:** 2024-04-30

**Authors:** David W. James, Marcos Quintela, Lisa Lucini, Nour Al Abdullah Al Kafri, Gareth D. Healey, Nicholas Jones, Kinza Younas, Adnan Bunkheila, Lavinia Margarit, Lewis W. Francis, Deyarina Gonzalez, R. Steven Conlan

**Affiliations:** ^1^ Swansea University Medical School, Swansea, United Kingdom; ^2^ Swansea Bay University Health Board, Swansea, United Kingdom; ^3^ Cwm Taf Morgannwg University Health Board, Bridgend, United Kingdom

**Keywords:** WT1, AR, transcription, epigenomics, endometrium, decidualization, polycystic ovary syndrome

## Abstract

Decidualisation, the process whereby endometrial stromal cells undergo morphological and functional transformation in preparation for trophoblast invasion, is often disrupted in women with polycystic ovary syndrome (PCOS) resulting in complications with pregnancy and/or infertility. The transcription factor Wilms tumour suppressor 1 (WT1) is a key regulator of the decidualization process, which is reduced in patients with PCOS, a complex condition characterized by increased expression of androgen receptor in endometrial cells and high presence of circulating androgens. Using genome-wide chromatin immunoprecipitation approaches on primary human endometrial stromal cells, we identify key genes regulated by WT1 during decidualization, including homeobox transcription factors which are important for regulating cell differentiation. Furthermore, we found that AR in PCOS patients binds to the same DNA regions as WT1 in samples from healthy endometrium, suggesting dysregulation of genes important to decidualisation pathways in PCOS endometrium due to competitive binding between WT1 and AR. Integrating RNA-seq and H3K4me3 and H3K27ac ChIP-seq metadata with our WT1/AR data, we identified a number of key genes involved in immune response and angiogenesis pathways that are dysregulated in PCOS patients. This is likely due to epigenetic alterations at distal enhancer regions allowing AR to recruit cofactors such as MAGEA11, and demonstrates the consequences of AR disruption of WT1 in PCOS endometrium.

## Background

Successful pregnancy relies on a delicate interplay of molecular and hormonal signals that transform the endometrial environment, making it suitable for blastocyst implantation and setting the stage for foetal development. Decidualization, the extensive cellular and molecular remodelling of endometrial stromal cells, which transform from fibroblast-like cells into large polygonal cells rich in cytoplasmic glycogen and lipids, is critical to this process (1). The ovarian steroids, in particular progesterone (P4), play key roles in decidualization and blastocyst implantation (2), driving a complex array of molecular events that are mediated by genes including Indian hedgehog (IHH) (3), Wigless-type MMTV integration site family (WNT) 4 (4), forkhead box O1 (FOXO1) (5), homeobox A10 (HOXA10) (6) and the Wilms Tumour suppressor gene (WT1) (7–9). Loss of function in any of these critical components can lead to impaired function and subsequent infertility.

Polycystic ovary syndrome (PCOS) is a highly prevalent disorder accounting for up to 44% of unexplained infertility cases and contributing to 21% of infertility in couples with ovulatory dysfunction. PCOS presents with symptoms including menstrual disturbance, hyperandrogenism and infertility, and is polygenic in nature, involving more than twenty associated genes involved in processes including secretion, molecular function, and extracellular matrix formation (10), though a definitive aetiology is still absent. Hyperandrogenism is at the core of PCOS and evidence from animal models indicates specific roles for testosterone in its pathogenicity. In healthy human endometrial stromal cells (hESCs), androgen receptor (AR) is present at low levels and functionally active in decidualization (11). However, in PCOS patients AR levels are significantly elevated, and in combination with higher levels of circulating androgens results in reproductive abnormalities (12). Spontaneous PCOS-like traits are observed in hyperandrogenic female non-human primate models, and exposure to dihydrotestosterone or early development of testosterone produces PCOS-like traits (13, 14). Furthermore, RNA-seq studies have demonstrated that gene networks involved in AR signalling are disrupted in PCOS patients (12), implying AR-mediated contributions to the pathogenesis of PCOS.

Previously we have demonstrated that stromal cell restricted WT1 is present in the endometrium of healthy individuals during the window of implantation, and accumulates at higher levels during decidualization, but crucially is absent in PCOS patients (7). This loss of WT1 coincides with an increase in the levels of activated AR, as well as MAGEA11, an AR coregulator, in PCOS (15). Using chromatin immunoprecipitation sequencing (ChIP-seq) we describe that in fertile hESCs, WT1 binds to cis-regulatory regions of genes exhibiting differential expression between secretory and proliferative phases in fertile endometrium. In PCOS hESCs exposed to dihydroxy testosterone (DHT), AR binds to DNA locations bound by WT1 in fertile hESCs. Colocalization of AR and WT1 binding sites likely results in dysregulation of these pathways in PCOS.

## Results

### MET events and WT1 levels are perturbed in endometrial stromal cells isolated from PCOS patients

**Figure 1:**
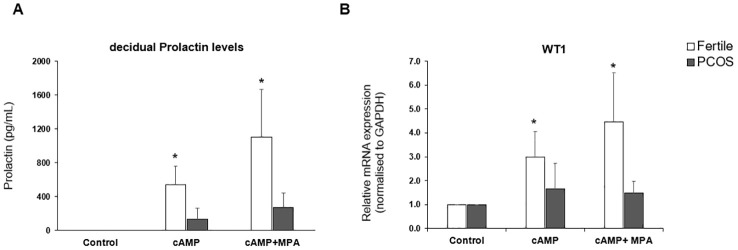
*In vitro* decidualization. Endometrial stromal cells were treated with medium or medium containing cAMP (0.5mM) or cAMP (0.5mM) and MPA (1 x 10-6 M) for 48hrs. Cells were lysed in RLT buffer before storage at -80°C and culture supernatant was collected and stored at -20°C. **(A)** decidual Prolactin levels of supernatant measured by ELISA in PCOS samples (n=10) vs fertile control (n=8). **(B)** Quantitative PCR for gene expression of WT1 mRNA normalised to GAPDH. Data presented as mean ± SD; for fertiles (n=8) and PCOS (n=7). Data was analysed by two-way ANOVA and Dunnett’s pairwise multiple comparison test, *p ≤ 0.05.

The transition of endometrial stromal cells from a mesenchymal to an epithelial phenotype (MET) is an essential prerequisite to blastocyst implantation (16, 17). hESCs were isolated from endometrial tissue biopsies and the response to a decidualization stimulus determined by monitoring the levels of prolactin. Decidualization was induced by incubating hESCs in the presence of cAMP (0.5mM) or cAMP (0.5 mM) plus MPA (1μM) for 48 h. *WT1* mRNA levels were significantly (*p* < 0.05) increased in hESCs isolated from fertile donors in response to cAMP +/- MPA, but not in endometrial PCOS cells (**Figure 1A**). Decidual prolactin (dPRL) levels were determined from samples cultured ex vivo and found to be increased in cells obtained from fertile individuals, whereas there was no response from PCOS derived stromal cells (**Figure 1B**).

### WT1 is recruited across the genome including to the regulatory regions of HOX and FOX genes following stromal cell decidualization

Having established that stromal cells derived from fertile donors were functionally active, the genome-wide targets of WT1 were evaluated to determine how the ‘gatekeeper’ role that we have previously proposed may manifest itself (7).

**Figure 2:**
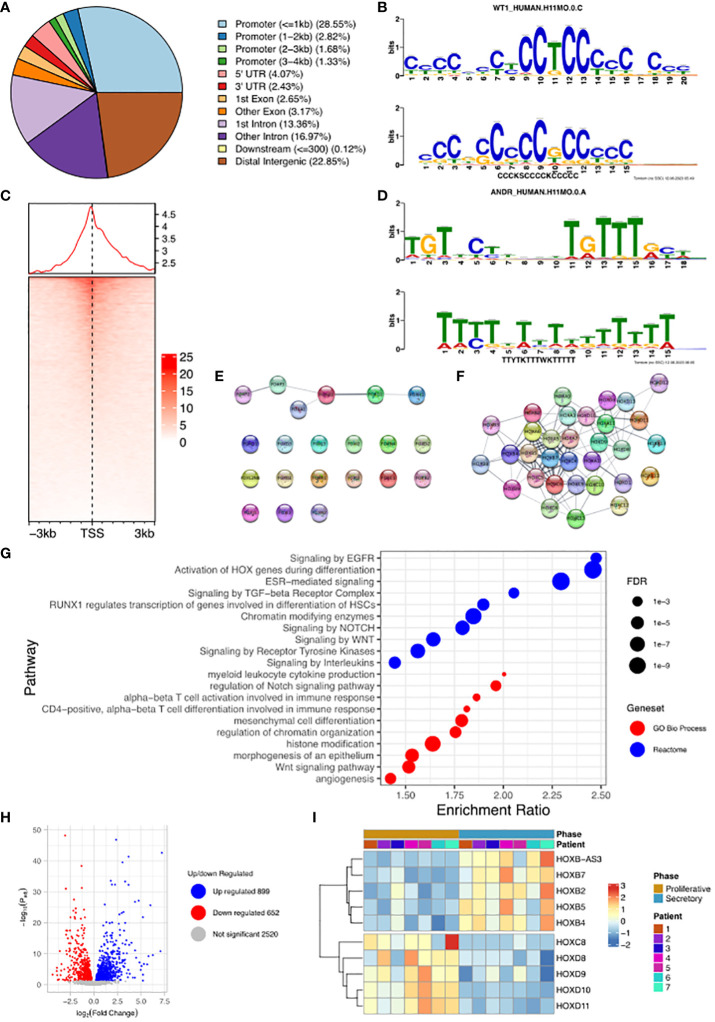
Analysis of WT1 binding in cAMP treated human endometrial stromal cells. **(A)** Percentage of WT1 peaks located in genomic feature types, **(B)** Binding MOTIF identified in WT1 binding regions which maps to WT1 binding motif (E = 5.35e-6), **(C)** Occupancy of WT1 binding peaks ±3kb around gene TSS’s, **(D)** Binding MOTIF identified in WT1 binding regions which maps to AR binding motif (E = 3.76e-2), **(E)** Network diagram of FOX genes with WT1 peaks in their promoter region, with edges indicating STRING DB protein-protein interactions, **(F)** Network diagram of HOX genes with WT1 peaks in their promoter region with edges indicating STRING DB protein-protein interactions, **(G)** ORA analysis results identifying significantly upregulated pathways for genes with WT1 peaks in their promoter regions (4kb upstream and 1kb downstream of TSS) from GO biological process, GO Cellular Component and Reactome gene sets, **(H)** Differential expression of mRNA in hESC, comparing proliferative phase vs secretory phase for genes with WT1 peaks in promoter regions from publicly available RNA-seq data (GSE86491), **(I)** Expression level of HOX genes with WT1 peaks in promoter regions exhibit significant differential expression between proliferative and secretory phase of menstrual cycle for 7 patients. WT1 ChiP-seq data deposited as GSE240055 in the NCBI GEO repository. RNA-seq expression data retrieved from NCBI GEO repository, accession GSE86491.

We undertook the mapping of genome-wide WT1 DNA-binding (deposited as GSE240055 in the NCBI GEO repository) in hESCs obtained from fertile patients treated with cAMP (0.5 mM). This *de novo* data was analysed and identified 19,417 called peaks (regions bound by WT1) (FDR < 0.05) revealing that WT1 DNA binding occurs across the genome (**Figure 2A**). DNA binding site motif analysis on genomic sequences contained within the WT1 binding peaks was used to find any consensus binding sites. As expected, MEME-ChIP identified a motif (E-value = 9.1e-47) that mapped to the WT1 motif (E-value = 5.35e-6) (18) (**Figure 2B**). Interestingly a second highly significant motif (E-value = 1.5e-81) was identified that mapped to the ANDR (AR) motif (E-value = 3.76e-2) (**Figure 2D**). The observation that AR binding sites are found in the immediate proximity of a high proportion of WT1 binding sites suggested that the two transcription factors could colocalise or that competitive exclusion of WT by AR could occur.

A more in-depth analysis identified 34% of peaks located within gene promoter regions close to the transcription start site (TSS, **Figure 2C**), with the majority of WT1 binding (29% of peaks) occurring within 1kb of the TSS suggesting that WT1 plays a role in transcription regulation through interactions with the core transcription machinery that assembles at these proximal promoter locations. WT1 was also seen to bind extensively within gene bodies, primarily within intron 1 (13% of peaks) and other introns (17% of peaks) suggesting a potential role in intron retention (19). There was also extensive WT1 binding at distal intergenic regions (23% of peaks, as defined by ChIPseeker) suggesting WT1 may function at enhancer sites.

WT1 binding was found to occur in the promoter regions of a large number of FOX genes (20 from 21 FOX genes, P-value < 0.001, **Figure 2E** and **Supplementary Table S1**) and homeobox (HOX) genes (32 from 34 HOX genes, P-value < 0.001, **Figure 2F**, **Supplementary Table S1**). HOX genes are essential for endometrial development and endometrial receptivity (20), and loss of FOX gene regulation has been linked PCOS (21), which suggests that the ‘gate keeper’ function of WT1 could be in the regulation of forkhead and homeobox genes during decidualization. Unsurprisingly therefore, over-representation analysis (ORA) (22) revealed ‘activation of HOX genes during differentiation’ (FDR < 0.001) as a major process, as well as ‘Estrogen dependent gene expression’ (FDR < 0.001), and Gene Ontology (GO) (23) biological process pathways revealed ‘Wnt signalling pathway’ (FDR < 0.001), ‘angiogenesis’ (FDR< 0.01) and ‘myeloid leukocyte cytokine production’ (FDR < 0.1) as significantly enriched (**Figure 2G**). During decidualization, endometrial stromal cells undergo morphological and functional changes, becoming mesenchymal, to allow implantation of the embryo (17, 24). Progression into the secretory phase of the menstrual cycle is governed by increases in the expression of estrogen (25). Wnt signalling is implicated in a number of implantation and decidualization events during mammalian pregnancy, and aberrant Wnt signalling negatively effects these processes (26, 27). Angiogenesis occurs during the secretory phase of the menstrual cycle, forming new blood vessels to supply nutrients in the case of a potential implantation event (28, 29). Many studies have shown the importance of interactions between endometrial cells including hESCs and leukocytes to regulate the processes associated with decidualization (30–32) including vascular remodelling of the decidua and angiogenesis (33).

The promoter regions of a large number (5,341) of DNA coding regions for mRNA, miRNAs and lncRNAs were bound by WT1 in our hESC decidualization model. We sought to determine if this corresponded to differential gene regulation using comparative RNA sequence data analysis. We accessed publicly available transcriptomic data RNA-seq (NCBI GEO repository accession GEO86491) from endometrium samples obtained from fertile patients and identified genes that were differentially expressed during decidualization (34). Comparing differentially expressed genes (DEGs) with WT1 gene targets determined that 598 were significantly upregulated and 836 significantly downregulated during decidualization (FDR < 0.05) (**Figure 2H**, **Supplementary Table S2**). These genes included several WT1 HOX genes targets, which displayed significant differential expression between proliferative and secretory phases. HOXB2, HOXB4, HOXB5 and HOXB7 were significantly up-regulated and HOXC8, HOXD8, HOXD9, HOXD10 and HOXD11 were significantly down-regulated in the secretory phase compared with the proliferative phase, and FOXO3 was downregulated in the secretory phase (**Figure 2I**, **Supplementary Table S1**). Consistent with our analysis, HOXD10 and HOXD11 have previously been found to be downregulated in the proliferative phase of the menstrual cycle (20). Additionally artificial induction of decidualization in mice elicits an increase in FOXO3 expression that is more pronounced at the implantation site in mouse uteri (35).

### AR is primarily located in enhancer regions in the genome of PCOS stromal cells

**Figure 3:**
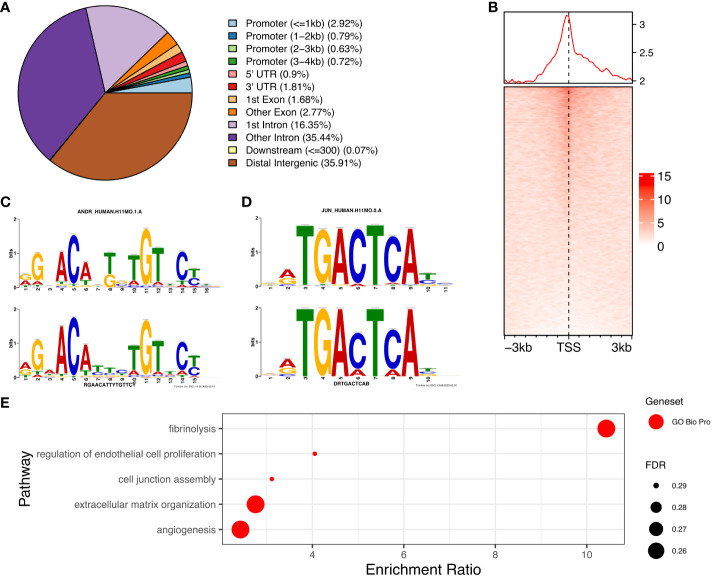
Analysis of AR binding in DHT treated human endometrial stromal cells. **(A)** Percentage of AR peaks located in genomic feature types, **(B)** Occupancy of AR binding peaks ±3kb around gene TSS, **(C)** Binding MOTIF identified in AR binding regions which maps to AR (E = 1.26e-2) and PRGR (E = 1.28e-2) binding motifs, **(D)** Binding MOTIF identified in AR binding regions which maps to JUN binding motif (E = 2.89e-8), **(E)** ORA analysis to identify significantly upregulated pathways for genes with AR peaks in their promoter regions (4kb upstream and 1kb downstream of TSS) from GO biological process, GO Cellular Component gene sets. AR ChiP-seq data deposited as GSE240055 in the NCBI GEO repository.

We determined the genome-wide localisation of AR (deposited as GSE240055 in the NCBI GEO repository) in hESC isolated from PCOS patients treated with DHT (15) to recapitulate events driven by hyperandrogenemia *in vivo*. Here we identified 12,017 significant AR peaks (FDR < 0.05%), of which only 5% were localised in gene promoter regions (**Figures 3A, C**), whereas 36% of peaks were located in distal intergenic (potential enhancer) regions and 51% were located in the first (16%) or other (35%) introns (**Figure 3A**). This suggests that the regulation of gene expression by AR in PCOS hESCs in response to elevated androgen levels is primarily via binding at putative enhancer sites, and that there could also be a role in intron retention (19) due to the other major proportion of AR binding being in introns. MOTIF analysis of DNA sequences within AR peaks identified a motif (E=2.7e-268) which mapped to both androgen receptor motif ANDR (E = 1.26e-2) (**Figure 3B**) and progesterone receptor motif PRGR (1.28e-6), and we have previously shown that the progesterone pathway is active in ovulatory PCOS patients (7).

The motif with second highest enrichment (E = 5.1e-89) mapped to the consensus binding site for JUN proteins (E = 2.89e-08) (**Figure 3D**), components of the AP-1 transcription activator complex that are known enhance AR activity and stimulate cellular proliferation in prostate cancer cells (36). ORA analysis of genes with AR peaks in promoter regions using the GO biological processes gene set, identified enrichment of ‘regulation of endothelial cell proliferation’, ‘extracellular matrix organization’ and ‘angiogenesis’ (FDR < 0.3) (**Figure 3E**), pathways known to be associated with the decidualization process (25). Comparing genes with AR peaks in their promoter regions with DEGs between proliferative and secretory phases revealed 83 genes which are up-regulated and 66 genes which are down-regulated, suggesting a specific AR mediated gene set profile that is dysregulated in PCOS (**Supplementary Figure S1**).

### WT1 and AR binding sites are co-located

**Figure 4:**
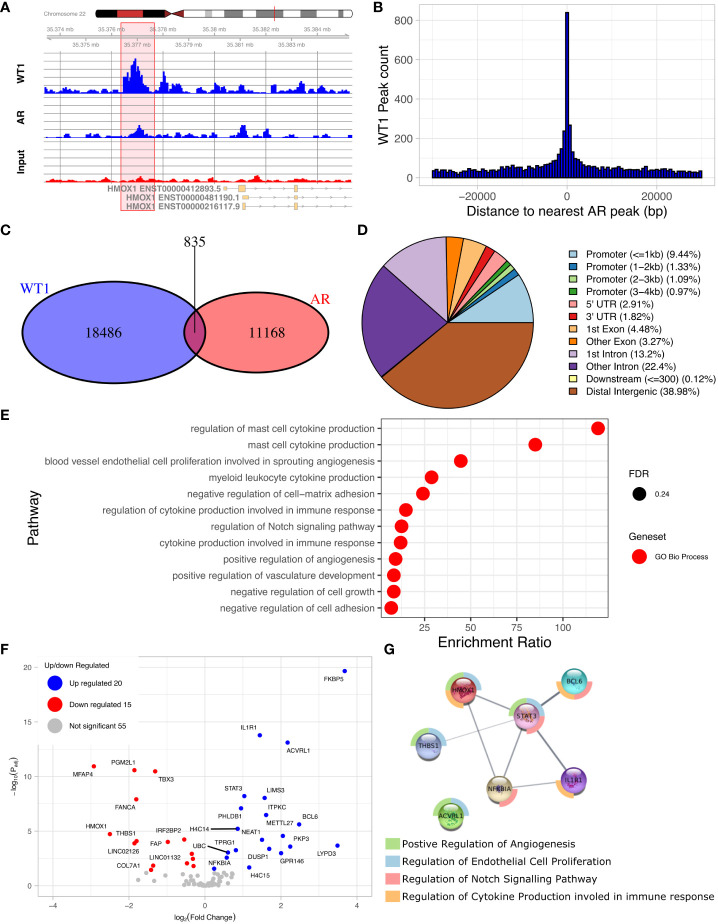
Overlaps of WT1 and AR ChIP-peaks in hESC treated with cAMP and DHT respectively. **(A)** Genome browser view showing co-located WT1 and AR ChIP peaks in the promoter region of HMOX1 gene, **(B)** Distance between WT1 peaks to the nearest AR peak, **(C)** Venn diagram showing number of WT1 peaks co-located with AR peaks, **(D)** Percentage of merged WT1/AR peaks located in genomic feature types, **(E)** ORA analysis to identify significantly upregulated pathways for genes with merged WT1/AR peaks in their promoter regions (4kb upstream and 1kb downstream of TSS) from GO biological process, GO Cellular Component and Reactome gene sets, **(F)** Differentially expressed genes (DEG) with merged WT1/AR peaks in promoter regions between proliferative and secretory phase of menstrual cycle, **(G)** Protein/protein interactions of selected DEGs with WT1/AR peaks in promoter regions between proliferative and secretory phase of the menstrual cycle and selected pathways which they are involved in. Outer ring colours indicate pathway. WT1 and AR ChiP-seq data deposited as GSE240055 in the NCBI GEO repository.

The discovery of an enriched AR motif in the WT1 peak set suggested that WT1 and AR binding sites could be co-located across the genome (**Figure 4A**, **Supplementary Figure S2**). We therefore measured the distance between each AR peak to the nearest WT1 peak (**Figure 4B**). We found that 7.0% of AR ChIP peaks directly overlapped with WT1 peaks (P < 2.2e-16) suggesting that the two transcription factors could mutually exclude one another from binding. Merging the WT1 and AR peak sets and keeping only overlapping WT1/AR peaks identified in 826 merged peaks (including multiple overlaps i.e. two WT1 peaks overlapping a single AR peak were considered as a single merged peak). Only 13% of merged peaks were localised in gene promoter regions, corresponding to 106 peaks and 100 genes (considering promoters may contain multiple peaks) (**Figure 4C**, **Supplementary Table S3**) including 15 lncRNAs and 10 miRNAs (**Supplementary Table S4**). The remaining 741 (87%) of WT1/AR merged peaks were located outside gene promoter regions (**Figure 4D**), with 39% located in distal intergenic (potential enhancer) regions (**Figure 4D**).

ORA analysis of shared WT1/AR targets revealed enrichment of GO biological function, including ‘blood vessel endothelial cell proliferation involved in sprouting angiogenesis’, ‘regulation of cytokine production involved in immune response’, ‘regulation of Notch signalling pathway’ and ‘positive regulation of angiogenesis’ (**Figure 4E**). Examination of RNA levels for DEGs with WT1/AR merged peaks in promoter regions using the Sigurgeirsson data set revealed 20 significantly up-regulated and 15 significantly down-regulated DEGs (**Figure 4F**) (34). FKBP5, which had the greatest increase in differential expression, has been shown to regulate decidualization through Ser473 dephosphorylation of AKT (37), and its dysregulation in rats is associated with aberrant PGR-targeted gene expression (38).

STRING database analysis (39) was then used to identify protein/protein interactions of significantly differentially expressed candidate WT1/AR target genes, and using networks visualization we revealed that several of these DEGs were present in the GO biological function pathways identified above (**Figures 4E, G**). This detailed network analysis identified interactions between proteins encoded by STAT3, BCL6, ILR1, HMOX1, dysregulation of which has been implicated in diseases of the female reproductive system (see discussion). Together this reinforces the likely importance of these proteins in the decidualization process, that their dysregulation, due to aberrant AR/WT1 binding in PCOS patients, may affect implantation success.

Finally, we considered the involvement of candidate miRNA and lncRNA WT1/AR targets (**Supplementary Table S4**) in biological processes and identified pathways upregulated by miRNAs to again include ‘Estrogen signalling pathway’ (P = 0.0015), ‘endometrial cancer’ (P = 0.0012), ‘Wnt signalling pathway’ (P = 0.0195), ‘Focal adhesion’ (P = 0.0002), ‘Adherens junction’ (P = 1.11e-16) and ‘FOXO signalling pathway’ (P = 0.008) (**Supplementary Figure S3**). Gene products targeted by lncRNAs with merged WT1/AR peaks include HOXA9, IGF1R, WT1, BCL6, FOXO1 and HOXA16 (**Supplementary Figure S4**).

### Co-Location of WT1 and AR corresponds with H3K4me3 and H3K27ac histone modifications in cis-regulatory elements

**Figure 5:**
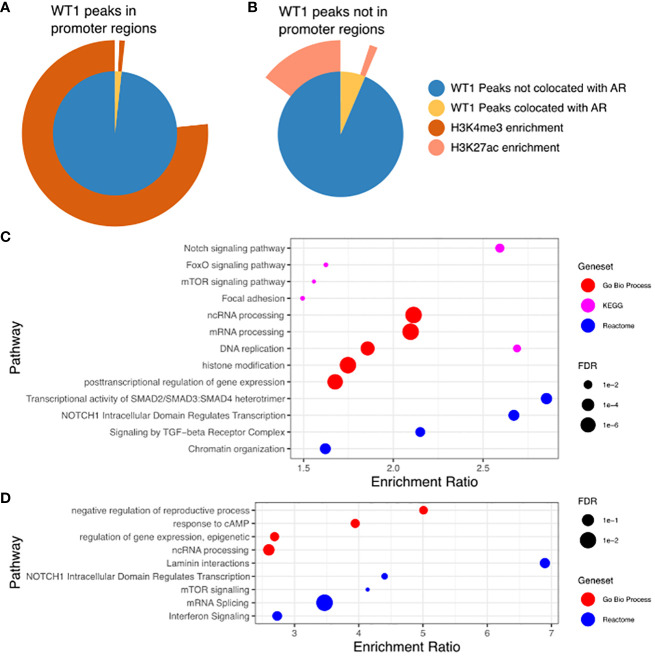
**(A)** WT1 peaks in gene promoter regions co-located with AR peaks and in H3K4me3 enriched regions. Inner pie chart shows peaks co-located with AR, outer ring shows WT1 and WT1/AR peaks in H3K4me3 enriched regions. **(B)** WT1 peaks outside of gene promoter regions co-located with AR peaks and in H3K27ac enriched regions. Inner pie chart shows peaks co-located with AR, outer ring shows WT1 and WT1/AR peaks in H3K27ac enriched regions. **(C)** Pathways enriched for genes regulated by enhancers containing WT1 peaks (gene/enhancer regulation annotations from GeneHancer database), **(D)** Pathways enriched for genes regulated by enhancers containing merged WT1/AR peaks (gene/enhancer regulation annotations from GeneHancer database). H3K27ac and H3K4me3 ChIP-seq data for decidualisation hESC from healthy human endometrium retrieved from NCBI GEO repository, accession GSE61793.

The proposed functional interplay between WT1 and AR occurs predominantly at distal intergenic regions, and therefore suggests a link to certain epigenetic marks that occur at gene enhancer sites. Enhancer regions are often located many 1000s of bp from gene TSS and are involved in the regulation of these genes through direct interactions with the core promoter via enhancer regions looping over to allow proteins recruited to that site to interact with the core transcription machinery (40). The histone marks H3K4me3 and H3K27ac are purported indicators of promoters and active enhancers respectively (41), with both marks enriched in regions of open chromatin, resulting in active gene transcription. To enable comparative meta-analysis between WT1 and AR binding events and the active histone marks, we exploited publicly available H3K4me3 and H3K27ac ChIP-seq data sets from endometrial stromal cells treated with cAMP and MPA to induce decidualization (GSE61793) (42). Of the 6,676 WT1 peaks located in gene promoter regions, 5,180 were co-located with H3K4me3 peaks (77.6%, P-value < 0.001 one-sided Fishers exact test) (see **Figure 5A**, **Supplementary Table S5**) indicating that WT1 binding is associated with active gene transcription in decidualization. Performing the same analysis on merged WT1/AR peaks, we found 59 were co-located with H3K4me3 peaks (63.2%, P-value < 0.001 one-sided Fishers exact test), (**Figure 5A**, **Supplementary Table S5**), further substantiating that any competitive binding between WT1 and AR in PCOS patients is likely to result in aberrant regulation of genes that are actively transcribed during decidualization. Performing ORA analysis on genes with both H3K4me3 and WT1 peaks within promoter regions identified a similar set of pathways as the previous analysis for genes with WT1 peaks in promoter regions. In contrast only 16.1% of WT1 peaks were located in H3K27ac enriched distal intergenic regions (P-value < 0.001 one-sided Fishers exact test) (**Figure 5B**, **Supplementary Table S5**), indicating that WT1 plays a limited function in regulating decidualization via these putative enhancers. From the WT1/AR merged peaks, 165 occupied H3K27ac enriched regions (22.9%, P-value < 0.001 one-sided Fishers exact test) (**Figure 5B**, **Supplementary Table S4**), implying that competitive binding of AR in WT1 binding regions affects putative enhancer activity in PCOS patients, resulting in aberrant gene regulation. Finally, we explored the GeneHancer database (43) to understand the biological functions regulated by WT1 and WT1/AR binding in distal intergenic regions and determined that 1,157 WT1 occupied locations (of the 4,437 sites identified in distal intergenic regions) were at defined enhancer locations known to regulate 4,117 unique genes (GeneHancer interaction score > 10), and potentially revealing 3,280 novel enhancer sites. Known enhancer sites corresponded to regulator regions for ‘Notch signalling’ (FDR < 0.01) and ‘FoxO signalling’ (FDR < 0.1) identified using pathway analysis (44) (**Figure 5C**), supporting direct ChiP-seq and comparative RNA-seq meta-analysis. Applying the same analysis to WT1/AR occupied distal intergenic regions, we identified a unique set of 153 annotated enhancers locations (from the 322 overlapping sites identified in distal intergenic regions), regulating 565 genes (GeneHancer interaction score > 10) that identified enriched pathways include ‘response to cAMP’ (P < 0.05) and ‘interferon signalling’ (P <0.001) (**Figure 5D**).

## Discussion

WT1 is normally expressed in the endometrial stromal cells during the window of implantation, where it appears to have a central role as a ‘gatekeeper’ in the process of decidualization (7, 45–47). To understand the underlying nature of this gatekeeper effect we conducted genome-wide mapping of WT1 binding in primary hESCs treated to stimulate effective decidualization. Our analysis revealed that indeed WT1 regulates the expression of cascades of genes important in correct endometrial function, and that this regulation occurs through both proximal and distal control of gene expression, often associated with epigenetic modifications localised to WT1 binding. RNA-seq data revealed that a significant proportion of putative WT1 target genes are differentially expressed between the secretory and proliferative phases of the menstrual cycle, providing strong evidence that WT1 plays an important role in implantation and decidualization, prerequisites to successful implantation. Furthermore, comparison with H3K4me3 and H3K27ac ChIP-seq datasets highlighted that WT1 binds in regions of open chromatin, in both promoter and enhancer regions in hESCs during decidualization, supporting the hypothesis that WT1 is a central regulator of gene expression during the window of implantation.

WT1 was recruited to 32 of the 34 promoter sites of HOX genes in hESC, suggesting that WT1 is an important determinant in the regulation of the HOX gene family during decidualization. HOXC8, and HOXD8, 9, 10 and 11 were downregulated in secretory phase, and conversely HOXB2, 4, 5, 7 were upregulated, demonstrating the functional consequences of WT1 binding to the regulatory regions of these genes. HOX genes are essential regulators of morphogenesis which is essential for endometrial development and endometrial receptivity (20). Interestingly HOXC10, HOXC11, HOXD10 and HOXD11, considered functionally redundant paralogs of HOXA10 and HOXA11 in development pathways, have been shown to perform distinct functions from their paralogs during decidualization, and are downregulated in the proliferative phase of the menstrual cycle (20). WT1 binding was observed in 20 of the 21 FOX gene promoter regions. The FOXA proteins are pioneer factors facilitating the opening of chromatin landscapes and subsequent promotion of tissue-specific transcription factor recruitment thus regulating cell specification and cell identity (48). FOXA1 and FOXA2 have been shown to control recruitment of glucocorticoid receptor in uterine cells (49), and regulation of implantation and endometrial remodelling (50). Knockdown experiments in mouse uterine stromal cells suggest that FOXO3 plays a role in regulating decidualisation factors (MMP9, MMP2, BMP2) and apoptosis-related factors (PARP, Bax, Bcl-2, Fas). In vivo knockdown of FOXO3 in mice has been associated with dysregulated apoptosis and reduced embryo numbers during early pregnancy (35). Our observations suggest that during hESC decidualization these pioneer factors are themselves governed by WT1.

### Crosstalk between WT1 and AR regulated pathways in PCOS patients

The loss of WT1 in PCOS patients coincides with an increase in the levels of activated AR (7, 15). Here we began to decipher how AR could dysregulate processes in PCOS patients that are regulated by WT1 in fertile women. We identified 106 promoter and 322 enhancers regions that contained overlapping WT1/AR peaks, and infer this is indicative of a competitive transcription factor binding between AR and WT1 within cis-regulatory regions in PCOS patients, governed by the relative abundance of AR within the cells. We identified a small number of putative WT1/AR regulated genes which exhibit significant differential expression between secretory and proliferative phases including FKBP5, which has the highest log_2_ fold-change. FKBP5 has been shown to regulate decidualization through Ser473 dephosphorylation of AKT (37), and its dysregulation in rats is associated with aberrant PR-targeted gene expression (38), which coincides with the recruitment to AR to PR sites in PCOS.

Network analysis of WT/AR targets identified interactions between STAT3, BCL6, ILR1, HMOX1, NFKBIA and THBS1; dysregulation of STAT3, BCL6, ILR1 and HMOX1 has been implicated in diseases of the female reproductive system, again demonstrating the validity of our approach. Ablation of STAT3 in murine models results in dysregulation of uterine epithelial remodelling, resulting in aberrant embryonic attachment (51), infertility (52) and implantation failure (53). BCL6 is involved in endometrial cell differentiation, migration, and invasion of trophoblastic cells (54). IL1R1 imbalance in ectopic endometrial tissue of women suffering from / with endometriosis results in heightened sensitivity to IL1 stimulation, which affects their ability to develop into host tissues for implantation (55). HMOX1 dysregulation has been implicated in PCOS (56), endometriosis (57), and *in-vitro* is down-regulated in endometrial stromal fibroblasts obtained from healthy patients in late secretory phase (58). Finally, PCOS patients exhibit significant differences in their immune cell population distribution and associated cytokine production, and display defective vascular remodelling of the endometrium (59). Our results indicate that WT1/AR crosstalk may affect genes that regulate immune-related and vascular remodelling-related pathways, resulting in reduced fertility in PCOS patients.

## Conclusion

The role of WT1 as a key regulator of decidualization is well known, however regulatory mechanisms have previously remained elusive. Here we show that WT1 regulates a large network of genes that are important for the functional and morphological changes in hESC cells. These genes include HOX and FOX transcription factors, which are known to regulate cellular differentiation.

Raised expression of circulating androgens and increased AR in hESC are key indicators of PCOS. Furthermore, WT1 expression in PCOS hESCs is reduced. Our study has shown a significant overlap between AR binding sites in PCOS hESCs and WT1 binding sites in fertile hESCs, revealing a mechanism which may result in irregularities in decidualization leading to symptoms commonly associated with PCOS such as infertility. Our results provide a rationale for development of strategies designed to re-introduce WT1 levels and/or reduce AR levels in endometrium of PCOS patients to re-establish regular decidual processes.

## Methods

### Stromal cell isolation

Primary hESCs were isolated from endometrial biopsies as previously described from patients of proven fertility and infertile patients diagnosed with anovulatory PCOS (47). Confluent hESC cells were washed twice in PBS and maintained in DMEM/F12 medium supplemented with 10% charcoal stripped media for 24 h prior to the start of the experiment. To induce decidualization, cells cultured in charcoal stripped media were treated with cAMP (0.5 mM), or cAMP (0.5 mM) and medroxyprogesterone acetate (MPA (1 μM) for 48 h. Decidualization was evaluated by analysing changes in cell morphology, and by measuring the secreted levels decidual PRL (dPRL) in the cell culture media, or with DHT (10^−8^ M), or DHT (10^−8^ M) + cAMP  (0.5 mM) for 48 h to simulate PCOS conditions.

### Measurement of secreted PRL in culture media by ELISA

Concentrations of secreted dPRL in cell culture media were measured using commercial ELISA kits according to the manufacturer’s instructions (DY682; R&D systems). Measurements were performed in triplicate.

### Chromatin immunoprecipitation

Chromatin immunoprecipitation (ChIP) was carried out as described previously following treatment with cAMP (0.5 mM) for fertile controls (15, 60). Following treatment, hESC cells were fixed using 1% formaldehyde solution (Sigma^®^), quenched with 2.5M Glycine (Sigma^®^) and centrifuged following Active Motif’s Epigenetic Services ChIP Cell Fixation Protocol instructions. The pellet was then sent to Active Motif for sequencing. An anti-WT1 antibody (ab89901 Abcam, UK) and anti-androgen Receptor antibody (ab9474, Abcam, UK) was used to probe for WT1 and AR-target region enrichment respectively.

### Analysis of ChIP-seq data

Fastq files were received from Active Motif^®^. Adaptor sequences were removed by Active Motif^®^. Prior to genome mapping, reads with more than 5 bases with Phred score less than 30 or reads containing undefined bases were removed (61). The sequencing quality of the remaining reads was determined using FASTQC (version 0.11.05, https://www.bioinformatics.babraham.ac.uk/projects/fastqc/) (**Supplementary Figure S5**). Trimmed sequencing reads were then mapped against the reference genome (hg38, GRCh38) using Bowtie 2 (version 2.2.9, http://bowtie-bio.sourceforge.net/bowtie2/index.shtml) with default parameters (62). Normalized strand coefficient (NSC) and relative strand correlation (RSC), indicators of ChIP-seq experiment signal to noise ratio, were assessed using phantompeakqualtools (63) (**Supplementary Figure S6**). SAM files were converted to BAM files, sorted and indexed using SAMTools (64). Reads mapping to DAC consensus excluded regions were removed (65). Peak calling for WT1 and AR enrichment above the input control using MACS software (1.4.2) (66) with p-value cut-off 1e-5 (66).

Fastq files of H3K4me3 and H3K27ac ChIP-seq samples on human endometrial tissue samples treated with cAMP were downloaded from the NCBI Geo repository from dataset GSE61793 using the NCBI SRA-toolkit. Quality control and mapping to the genome were performed in the same way as with transcription factor ChIP-seq samples. Peak calling of H3K4me3 samples was performed using MACS2 software, with broad peak option, and qvalue cut-offs of 5e-2 and 1e-1 for narrow peak and broad peak regions respectively. Peak calling for H3K27ac samples was performed using epic2 (version 0.041, https://github.com/biocore-ntnu/epic2) with FDR cut-off of 0.05 (67). Bigwig files were generated from BAM files using Deeptools (version 3.3.1) (68).

P-values of overlapping peaks from WT1, AR and histone mark datasets were calculated using one-sided Fisher’s exact test. For each pairwise comparison between peak sets, the genome was divided into equal length bins of 654 bp long (corresponding to the mean length of WT1 and AR peaks). A two-by-two contingency table was constructed with upper left containing the number of bins without any peaks, lower left containing the number of bins exclusively containing peaks from the first peak set, the upper right bin containing the number of bins exclusively containing peaks from the second peak set and the lower right cell containing the number of bins with peaks from both peak set 1 and peak set 1. P-values were calculated from the contingency table using the fisher.test R function (69).

### RNA-seq analysis

RNA-seq raw count data, containing sequencing results from seven paired healthy endometrial tissue samples taken in proliferative and secretory phases of the menstrual cycle were downloaded from the NCBI GEO repository GSE86491 (34). Differential analysis was performed comparing mRNA expression in secretory to proliferative phases using DESeq2 (70).

### Data analysis

Data analysis was performed using R (version 3.6.3) (69). Overlapping peak between samples were evaluated and merged peak sets were generated using the findOverlapsOfPeaks function in the ChIPPeakAnno (71) Bioconductor R package. Pairs of peaks defined as overlapping contained at least one common genomic coordinate. Peaks were assigned to genomic regions using the ChIPPeakAnno function assignChromosomeRegion. Genes were associated with peaks using the findOverlaps function in the GenomicRanges R Bioconductor package (72), to determine genes within 5000bp of peaks. Gene overlaps were assessed using the intersect function in R. Gene coordinates were obtained from EnsDb.Hsapiens.v86 R Bioconductor package (73). Enrichment heatmaps were created using the EnrichedHeatmap Bioconductor package (74). Genome browser plots were created using GViz R package (75). Venn diagrams were created using the ChIPPeakAnno function makeVennDiagram for overlapping peaks and the R VennDiagram package for overlapping genes. All other graphics were created using ggplot2 (76) in R. Motif analysis was performed using the MEME Suite MEME-ChIP web application (http://meme-suite.org/tools/meme-chip) (77). Multiple Expectation maximization for Motif Elicitation (MEME) (version 5.1.1) and Discriminative Regular Expression Motif Elicitation (DREME) software’s (version 5.1.1) were used for motif discovery, with threshold of E ≤ 0.05. HOCOMOCO Human (v11 CORE) input motif set was used. Over-representation analysis was performed using WebGestaltR R package (78). The ‘BH’ FDR method was used, with an FDR threshold of 0.05. ORA analysis was performed for GO Biological process, GO cellular component (23), and Reactome (79) gene sets. Network analysis to identify protein/protein interactions was performed using the STRING DB plugin (39) for Cytoscape (80). Enhancer locations and enhancer/gene regulatory relationships were obtained from the GeneHancer database (43). Genes and pathways associated with miRNAs were identified using DIANA-mirPath v.3 web application (81). Genes and pathways associated with lncRNAs were identified using LncSEA web application (82).

### Gene expression analysis

Gene expression analysis was performed according to the MIQE guidelines (83). Total RNA was isolated from cells following lysis in RLT buffer using the RNeasy Mini kit (Qiagen, Manchester, UK), according to the manufacturer’s instructions. Reverse transcription of 1 μg mRNA was performed in a 20 μl reaction volume using the High Capacity cDNA reverse transcription kit (Thermo Fisher), according to the manufacturer’s instructions. Quantitative PCR primers were designed using the Primer-BLAST primer design software (http://www.ncbi.nlm.nih.gov/tools/primer-blast/) and validated by BLAST analysis against the *Homo sapiens* (taxid:9606) Refseq mRNA database. Primers and *GAPDH* reference gene primers were obtained from Sigma–Aldrich: *WT1* forward, 5’-CTATTCGCAATCAGGGTTACAG-3’, reverse, 5’-CATGCTTGAATGAGTGGTTGG-3’; *GAPDH* forward, 5’-GTCCACTGGCGTCTTCAC-3’, reverse, 5’-CTTGAGGCTGTTGTCATACTT-3’. Quantitative PCR was performed in a 10 μl reaction volume comprising 1 × iTaq Universal SYBR Green Supermix (Bio-Rad) with primers added in nuclease-free water to a final concentration of 0.4 mM and 2 μl of cDNA. Thermal cycling parameters were as follows: one cycle of 95°C for 5 min, followed by 40 cycles of 95°C for 10 s and 60°C for 60 s. The relative quantification method was employed to quantify target gene mRNA within samples (84). To generate standard curves, total RNA extracted from cells was reverse transcribed to cDNA, as described. Ten-fold serial dilutions of this reference cDNA were prepared (neat to 1 × 10^-3^) in nuclease-free water (Qiagen). For each sample, target and reference gene mRNA abundance was determined from the appropriate standard curve (quantification cycle, Cq). Changes in mRNA abundance between samples were then determined from the ratio of the target gene Cq to the reference gene Cq.

### Statistical analysis

Statistical analyses were performed using IBM SPSS Statistics 22 with biological replicate as the experimental unit. Initially the data were tested for homogeneity, and log or square root transformed if appropriate. Parametric data were analysed by analysis of variance (ANOVA) using Dunnett’s pairwise multiple comparison t-test for individual group comparisons. Data are presented as mean with standard deviation (SD), *p* < 0.05 was considered statistically significant, and the number of independent experiments is stated in the figure legends.

Statistical analysis of enrichment overlapping gene or peak sets was performed in R using the phyper function (which performs one-sided Fisher’s exact test).

## Data Availability

The datasets presented in this study can be found in online repositories. The names of the repository/repositories and accession number(s) can be found below: https://www.ncbi.nlm.nih.gov/, GSE240055.
