# Corrigendum: Homeobox regulator Wilms Tumour 1 is displaced by androgen receptor at cis-regulatory elements in the endometrium of PCOS patients

**DOI:** 10.3389/fendo.2024.1450375

**Published:** 2024-09-27

**Authors:** David W. James, Marcos Quintela, Lisa Lucini, Nour Al Abdullah Al Kafri, Gareth D. Healey, Nicholas Jones, Kinza Younas, Adnan Bunkheila, Lavinia Margarit, Lewis W. Francis, Deyarina Gonzalez, R. Steven Conlan

**Affiliations:** ^1^ Swansea University Medical School, Swansea, United Kingdom; ^2^ Swansea Bay University Health Board, Swansea, United Kingdom; ^3^ Cwm Taf Morgannwg University Health Board, Bridgend, United Kingdom

**Keywords:** WT1, AR, transcription, epigenomics, endometrium, decidualization, polycystic ovary syndrome

In the published article, there was an error in the author list, and author Nicholas Jones was erroneously excluded. Author Nour Al Abdullah Al Kafri was incorrectly listed as Noor K. Alkafri. The corrected author list appears below.

“David W. James, Marcos Quintela, Lisa Lucini, Nour Al Abdullah Al Kafri, Gareth D. Healey, Nicholas Jones, Kinza Younas, Adnan Bunkheila, Lavinia Margarit, Lewis W. Francis, Deyarina Gonzalez, R. Steven Conlan”

In the published article, there was an error in the legend for **Figure 1** as published. The figure legend included the treatment group cAMP+DHT that is not part of this manuscript. Also, the incorrect dataset and number of patient samples was used.

The corrected legend appears below.

In the published article, there was an error in **Figure 1** as published. Wrong dataset and number of patients used. The corrected **Figure 1** and its caption “*In vitro* decidualization” appear below.

**Figure 1:**
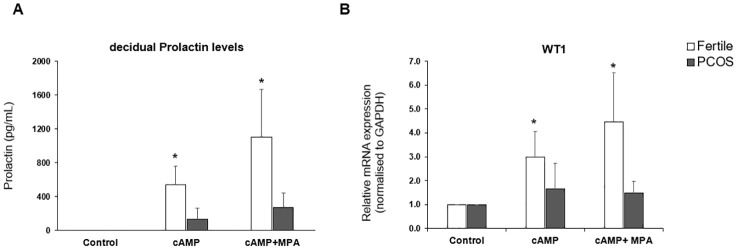
*In vitro* decidualization. Endometrial stromal cells were treated with medium or medium containing cAMP (0.5mM) or cAMP (0.5mM) and MPA (1 x 10-6 M) for 48hrs. Cells were lysed in RLT buffer before storage at -80°C and culture supernatant was collected and stored at -20°C. **(A)** decidual Prolactin levels of supernatant measured by ELISA in PCOS samples (n=10) vs fertile control (n=8). **(B)** Quantitative PCR for gene expression of WT1 mRNA normalised to GAPDH. Data presented as mean ± SD; for fertiles (n=8) and PCOS (n=7). Data was analysed by two-way ANOVA and Dunnett’s pairwise multiple comparison test, *p ≤ 0.05.

The authors apologize for this error and state that this does not change the scientific conclusions of the article in any way. The original article has been updated.

