# Supplementary material for: Homeobox regulator Wilms Tumour 1 is displaced by androgen receptor at cis-regulatory elements in the endometrium of PCOS patients
Source: Front Endocrinol (Lausanne). 2024 Apr 30;15:1368494. doi: 10.3389/fendo.2024.1368494 (PMC11091321; doi:10.3389/fendo.2024.1368494)
Supplement: Supplementary file 1 [file DataSheet1.docx]

**Supplementary Information**

**Results**

*WT1 is recruited across the genome including to the regulatory regions of HOX and FOX genes following stromal cell decidualisation*

Table 1 HOX and FOX genes with WT1 peaks in promoter regions

| **HOX Genes** | **FOX Genes** |
| --- | --- |
| HOXA1 | FOXA1 |
| HOXA10-AS | FOXB1 |
| HOXA11 | FOXB2 |
| HOXA11-AS | FOXC1 |
| HOXA13 | FOXD1 |
| HOXA3 | FOXD2 |
| HOXA5 | FOXD2-AS1 |
| HOXA6 | FOXD3 |
| HOXA7 | FOXD3-AS1 |
| HOXA9 | FOXG1 |
| HOXB-AS3 | FOXH1 |
| HOXB13 | FOXI2 |
| HOXB2 | FOXI3 |
| HOXB3 | FOXJ1 |
| HOXB4 | FOXJ2 |
| HOXB5 | FOXJ3 |
| HOXB7 | FOXL2NB |
| HOXB8 | FOXN2 |
| HOXB9 | FOXN3-AS1 |
| HOXC-AS2 | FOXN4 |
| HOXC-AS3 | FOXO3 |
| HOXC10 | FOXP2 |
| HOXC12 | FOXP3 |
| HOXC13 | FOXQ1 |
| HOXC13-AS |  |
| HOXC4 |  |
| HOXC5 |  |
| HOXC6 |  |
| HOXC8 |  |
| HOXC9 |  |
| HOXD1 |  |
| HOXD10 |  |
| HOXD11 |  |
| HOXD12 |  |
| HOXD13 |  |
| HOXD3 |  |
| HOXD8 |  |
| HOXD9 |  |

Table 2 WT1 gene significantly upregulated and downregulated during decidualization (FDR < 0.05)

| **Upregulated** | **Downregulated** |
| --- | --- |
| LHFPL3 | YEATS2 |
| CXCL14 | DCAF7 |
| FEZF1-AS1 | USP8 |
| SLC30A2 | ATRN |
| CYP4B1 | GPATCH2L |
| LINC02884 | SENP6 |
| EBF2 | UBP1 |
| IRX3 | PCNX4 |
| PAX9 | PSMA3-AS1 |
| COMP | METTL16 |
| C2CD4B | ZNF250 |
| ATG9B | SCAF8 |
| RGS9BP | SEPTIN2 |
| SHISA3 | TRIM44 |
| SLC3A1 | KIAA2026 |
| SLC46A2 | ADAM17 |
| FBXL16 | ZNF407 |
| CILP | ADAR |
| PNMT | TTLL5 |
| CEMP1 | CHD8 |
| PTGDS | DNM1L |
| IRX5 | ZFAND4 |
| PRUNE2 | TP53BP2 |
| FKBP5 | CHD9 |
| ADAMTS15 | QKI |
| TFPI2 | RBM4 |
| TSKS | EDRF1 |
| CLDN4 | TRNT1 |
| LYPD3 | ZZZ3 |
| CEBPD | PLEKHA8 |
| PPP1R3G | PPP1R12A |
| PCSK6 | ZNF131 |
| PLCL1 | USP37 |
| INSRR | CNEP1R1 |
| BARX2 | DLEU2 |
| LINC02289 | IPO7 |
| GPBAR1 | CDC40 |
| RASD1 | MEIS2 |
|  | MTAP |
| IP6K3 | SRSF3 |
| ANKRD33B | RNF139 |
| LRRC1 | CNOT2 |
| ANG | HOMER1 |
| PKD1-AS1 | SMARCC1 |
| SMIM5 | KNOP1 |
| GCNT4 | PIBF1 |
| SLC2A8 | PHIP |
| CLEC3B | ZNF33A |
| SLCO4A1 | E2F6 |
|  | LNPEP |
| LINC01750 | ADO |
| CD55 | TSTD2 |
| LINC02043 | TNPO1 |
| S100A1 | ANP32A |
| CKB | REV1 |
| TMEM184A | ZNF397 |
| IRS2 | THADA |
| POPDC3 | CROCCP3 |
| HPSE | HNRNPLL |
| LRATD2 | GGPS1 |
| FMO5 | DHX35 |
| TMEM63C | G3BP1 |
| AQP3 | TPR |
| KLF4 | KPNA3 |
| CCN3 | IFT52 |
| SLC43A1 | FBXO11 |
| PYY | CPSF3 |
| DCXR | STMP1 |
| NFIL3 | HNRNPH3 |
| WHRN | CREB1 |
| ZDHHC11B | MYNN |
| PGM5P4-AS1 | PPIG |
| CLDN3 | NBAS |
| KLF6 | MIRLET7A1HG |
| CITED2 | ANKIB1 |
| NR4A2 | BMPR2 |
| BCL6 | TMX4 |
| CRYAB | ZNF345 |
| RPRM | ZNF362 |
| MAFF | NAGK |
| CASZ1 | DCAF13 |
| SIK1 | CWC22 |
| LRRC73 | SCRN1 |
| MOGAT1 | TRMT1L |
| PER1 | CAND1 |
| GDF15 | RPAIN |
| PC | SEL1L |
| EVA1C | HNRNPUL2 |
| NDRG1 | CTDSPL2 |
| SLC26A8 | SAE1 |
| SLC22A3 | COX20 |
| DCDC2B | CAPRIN2 |
| ITGB8 | NDUFAF6 |
| DMRTA1 | SAYSD1 |
| PKP3 | APMAP |
| TMEM92 | KLHL22 |
| TOB1 | KDM4A |
| HEY1 | PNMA1 |
| PPP1R1B | NCBP1 |
| IZUMO4 | HMCES |
| TENT5A | SP3 |
| NNMT | B4GAT1 |
| ACVRL1 | ZFP91 |
| SH3BGRL3 | TCEANC2 |
| GGT1 | TMEM185B |
| PNPLA2 | ZFP36L1 |
| TPPP3 | REEP3 |
| HOXB-AS3 | TADA1 |
| GJC2 | DPH5 |
| MTARC1 | KDM5B |
| GABARAPL1 | CEP76 |
| ALDH6A1 | TCERG1 |
| WDR86-AS1 | L2HGDH |
| METTL27 | POLR2B |
| TOB1-AS1 | AHI1 |
| PSAT1 | DHX9 |
| TMEM120A | EMX2OS |
| KIFC2 | SMAD5 |
| REEP6 | ING1 |
| TOM1 | CPSF2 |
| CNDP2 | FRS2 |
| GPR146 | ZNF484 |
| ZDHHC12 | MAN2A1 |
| TLCD1 | CASP2 |
| LCN12 | PTMA |
| SLC25A29 | KCNQ1OT1 |
| H2AC6 | FNDC3A |
| HSPB1 | HNRNPD |
| NABP1 | APOBEC3C |
| ZFP36 | CHD4 |
| SP5 | TMEM123 |
| GALNT4 | ZNF616 |
| BTG2 | ZNF33B |
| GARNL3 | ZNF790 |
| ANXA4 | HNRNPA1 |
| IL6ST | VPS13B |
| KLF9 | GTF2IRD2B |
| C1orf21 | LANCL2 |
| ATP1A1 | GMPS |
| FAM13A | GXYLT1 |
| SPINT1 | IFT88 |
| RRAS | ZNF496 |
| TUB-AS1 | HERC6 |
| EZR | CNOT7 |
| JUNB | RNF168 |
| ARRDC1 | TNRC6B |
| ROPN1L | LYRM4 |
| UCKL1-AS1 | SP4 |
| MRPS2 | GOLM2 |
| ARHGAP20 | SSH1 |
| TIPARP | SOCS5 |
| DDIT4 | M6PR |
| CHRD | BTN3A1 |
| ADAMTS5 | PPIL4 |
| AK7 | CORO1C |
| DSCAML1 | HOXD8 |
| ADGRD1 | RAB34 |
| WARS1 | RBBP4 |
| TRAPPC6A | PMS1 |
| PER2 | EMSY |
| MST1R | TMEM65 |
| FLVCR2 | RSRC1 |
| IFNGR1 | SMC3 |
| ZBTB7B | CTCF |
| PRKAG2 | GLS |
| CSRNP1 | PLPP1 |
| ELL2 | SYNCRIP |
| SQLE | ARL3 |
| CDC34 | HMGB1 |
| MPC1 | POC5 |
| TRABD | OSBPL8 |
| PALM | WASF1 |
| LINC00921 | BLMH |
| GATA6 | ZEB2 |
| TBC1D8 | CUL4B |
| GAD1 | ZNF84 |
| HAND2 | KIAA0753 |
| KCND3 | USP1 |
| PIM3 | ILF3 |
| DUSP1 | RIC1 |
| GCAT | HDGFL3 |
| SH2D3A | MRPL1 |
| ACKR2 | HERPUD2-AS1 |
| LPCAT3 | HYKK |
| SMIM6 | GTF2IRD2 |
| ZBTB7C | TPGS2 |
| CARD9 | EPM2AIP1 |
| ADISSP | DENND5B |
| CALHM2 | ITPKB |
| BCAT2 | MYLIP |
| TLL1 | ANKMY2 |
| FLT3LG | DDX20 |
| PNKD | ZDHHC17 |
| CIB1 | CBX3 |
| HMGCR | GTDC1 |
| MKNK2 | SMC1A |
| TUBB4B | HECTD2 |
| ARLNC1 | LINC01410 |
| ITPKC | BZW1 |
| SECISBP2L | PTPN21 |
| ANKRD37 | ACAA2 |
| DUSP23 | TRAM2-AS1 |
| RPUSD1 | ZNF32 |
| ACSL4 | RBM4B |
| MUC1 | ANO6 |
| SH3GLB2 | KDM1A |
| LIMS3 | SRSF1 |
| ELOB | SMARCA5 |
| PEMT | EPM2A |
| AVPI1 | CSGALNACT2 |
| PTPN3 | ZNF649 |
| FBXW5 | FADS1 |
| NDRG2 | HNRNPA0 |
| HSF4 | SLC18B1 |
| LIMS4 | ITGAV |
| ZBTB42 | ZNF267 |
| SPAG6 | IRF2BP2 |
| SPATA25 | FGFR1 |
| CORO6 | STT3B |
| BCL3 | SIPA1L1 |
| NEAT1 | BAZ1B |
| TNS2 | ATP6V1E2 |
| POLD4 | APP |
| PRRT2 | BMT2 |
| SPINT2 | HEXB |
| STBD1 | POLE3 |
| CYB561 | ASPH |
| FOSL2 | FLVCR1 |
| AP1S3 | PTDSS1 |
| GPR108 | BLOC1S6 |
| WIPI1 | PASK |
| ALKBH7 | CYP2U1 |
| MTHFR | ZNF786 |
| GNB2 | LRRC20 |
| COL5A3 | H2BC5 |
| IL1R1 | TANC1 |
| AGPAT2 | INTS7 |
| FLNB | ACACB |
| ADAMTSL4 | ALMS1 |
| SERPING1 | CEP57 |
| SLC25A38 | TVP23C |
| HAGH | CDCA4 |
| KCNIP1 | CCDC77 |
| TMTC1 | RIPK2-DT |
| S100A10 | MAGI2-AS3 |
| LINC01124 | NEIL2 |
| SSNA1 | VOPP1 |
| FKBP2 | TP53I11 |
| NME3 | NR1D2 |
| THAP4 | ZNF793 |
| SLC6A6 | CCT6B |
| YBX3 | MICOS10 |
| SQOR | N4BP2 |
| ARHGEF35 | B3GNT9 |
| GUK1 | BCL9 |
| ATP7B | CBL |
| MIR200CHG | PBX3 |
| CLPP | THNSL1 |
| UAP1 | MLLT3 |
| PLEC | NCAPD3 |
| MATN2 | ARHGEF40 |
| FTH1 | FBN1 |
| CST3 | VIM |
| ZC3H12A | TP53BP1 |
| OSER1 | RAP2B |
| MYL3 | DNMT1 |
| MTR | ITPR2 |
| ADAMTS1 | TCAF1 |
| GPX4 | HSPH1 |
| STUB1 | MAPRE2 |
| CNPPD1 | JARID2 |
| LRP10 | ZFTA |
| P4HA1 | KIF22 |
| PRDX5 | FNDC3B |
| SERINC5 | VEZF1 |
| SNAPC2 | MAP1A |
| CRB3 | CENPJ |
| WDR97 | RFX3 |
| ID4 | EYA2 |
| DCPS | OSBPL1A |
| TP53INP1 | TIGD1 |
| EZH1 | R3HDM1 |
| RASA4B | NES |
| ITGB8-AS1 | DONSON |
| UBALD1 | STAT1 |
| LNC-LBCS | ZNF860 |
| FRAT2 | DCLK2 |
| MYC | ERV3-1 |
| RSRP1 | KIRREL1 |
| C19orf25 | SHF |
| TLE1 | TMEM200B |
| TPCN1 | TFAM |
| FGF14-IT1 | CNN3 |
| DVL1 | TMEM237 |
| IL5RA | TRDMT1 |
| PSPH | SPRED2 |
| RAB40C | JMJD1C |
| HS6ST1 | PPP1CC |
| CYP4F11 | KLF10 |
| RNPEPL1 | KHDRBS1 |
| BSG | PLK2 |
| MAPK12 | CCDC171 |
| TSC2 | CALML4 |
| RNF125 | MAF |
| PTGER2 | NFYA |
| JUN | NUCKS1 |
| ATP6V0B | SHROOM4 |
| DYNLRB1 | ABI2 |
| NPDC1 | LRRN4CL |
| MAP3K8 | DIPK2A |
| TAGLN2 | RNF24 |
| AHNAK | NEMP1 |
| SH3BGRL2 | LIX1L |
| PRDM8 | BCL2L11 |
| MERTK | TOP2B |
| ANKRD23 | TSHZ3 |
| HOXB7 | LPP-AS2 |
| DUSP4 | PCDH9 |
| REV3L | RNF144A |
| HERPUD1 | PLEKHA8P1 |
| SREBF1 | OSGEPL1 |
| CARMIL1 | BMI1 |
| MAF1 | PTCH2 |
| GMNN | CNTRL |
| SHROOM1 | DST |
| KIAA0513 | HEG1 |
| NEK6 | FCHSD2 |
| ALDH4A1 | FBXL19-AS1 |
| ATG2A | HOXD9 |
| COX8A | AUTS2 |
| KLHL21 | SH2B3 |
| CD151 | LINC00665 |
| TMUB1 | TIGAR |
| SLC4A7 | NR2F2 |
| CYSTM1 | NASP |
| TSPAN13 | ME3 |
| S100A13 | PDE10A |
| JPT2 | SKA2 |
| TST | AGAP2 |
| KBTBD3 | IGFBP4 |
| CDIPT | CHD3 |
| LRRC41 | SDCBP2-AS1 |
| NUP153 | PATL2 |
| DNAJC15 | PANK1 |
| NHERF2 | ZNF703 |
| NEURL2 | TUT4 |
| H4C15 | ZFP37 |
| HPS6 | ACTL6A |
| NADK | RGS10 |
| BACH2 | TTC3 |
| UCKL1 | TOX |
| HIPK3 | SUV39H2 |
| HSD11B2 | TBC1D31 |
| SNX32 | GNAO1 |
| AGTRAP | HIC1 |
| EOLA1 | FMNL3 |
| FAM3A | PARP8 |
| CPEB2 | CALU |
| PITPNM1 | NXPE3 |
| NAMPT | USP49 |
| GPR137 | RMND1 |
| ULK1 | MDK |
| TBX2-AS1 | SYDE2 |
| NHERF1 | EZH2 |
| LNX2 | RAB23 |
| SLC50A1 | RUNX1T1 |
| AKAP12 | IGFBP7 |
| ADAM15 | SLFN11 |
| TEKT1 | GPRASP2 |
| CEP126 | QSER1 |
| ALDOA | PARD6G |
| SMIM29 | PIK3CG |
| POLR2L | ZNF117 |
| COQ8A | FAM72B |
| WBP2 | LINC01482 |
| NFE2L1 | MSH2 |
| CDR2L | EMX2 |
| OSBPL5 | NCAPD2 |
| PTH1R | STOX2 |
| COL18A1 | FKBP7 |
| ELOVL1 | TEAD2 |
| CHPF | CEP135 |
| JOSD1 | MCM7 |
| ST3GAL1 | ZNF503 |
| C7orf57 | FKBP14 |
| KMT5C | H2AC21 |
| GYS1 | POC1A |
| SAMD13 | WASF3 |
|  | RCBTB2 |
| NUDT16L1 | ZNF713 |
| PLA2G7 | ST3GAL2 |
| SYNE3 | B4GAT1-DT |
| ASPSCR1 | HMGN2 |
| CNNM3 | PIPOX |
| AIP | CMTM6 |
| CHMP2A | COL6A3 |
| CEP170B | CCDC18 |
| D2HGDH | A2M-AS1 |
| ROM1 | TEF |
| HDAC5 | HOXD10 |
| TMEM187 | SLC16A2 |
| AAMP | RFX5 |
| PITPNM2 | CCDC88A |
| FAM3C | HIPK2 |
| PLEKHG3 | ARRB2 |
| WDR24 | PSIP1 |
| CTDSP1 | ARMT1 |
| CCDC183-AS1 | TFAP2C |
| FOXN3-AS1 | CBX2 |
| SF3B5 | PDE7A |
| STAT3 | FAM72A |
| CLXN | TMEM97 |
| RERG | FAP |
| ORMDL2 | EXTL2 |
| FARSA | ZNF462 |
| TPGS1 | MYH10 |
| TP53INP2 | VIRMA-DT |
| STAT6 | LRRC8B |
| TRADD | SFXN2 |
| GOT1 | PPAT |
| LZTR1 | MB21D2 |
| ERN1 | OSBPL6 |
| UBE2M | H2AZ1 |
| SERTAD1 | WNT2 |
| TRIP10 | STMN1 |
| DGKD | DUSP7 |
| CHMP6 | GFI1 |
| RAB11B-AS1 | SLC39A6 |
| NAPA | DOCK4 |
| SRRM2-AS1 | HES1 |
| SLC2A1 | H2BC8 |
| SPRYD3 | EFNB3 |
| ATP11A | MECOM |
| PRXL2A | PTENP1 |
| FAIM | BICD1 |
| CYB5R2 | ACVR1 |
| DDI2 | BEND6 |
| UBXN6 | ARL15 |
| IL15RA | NTN1 |
| TIMM13 | FMN1 |
| PIAS4 | METTL21A |
| TMSB10 | COL5A1 |
| PPP2R5A | TRIM45 |
| TBC1D16 | PRIM1 |
| CLCF1 | PLAU |
| XPO6 | PHGDH |
| SPAG8 | CRMP1 |
| TAPBPL | H2AC7 |
| UBL4A | INTS6L |
| NR2F6 | PTCH1 |
| MRPL55 | ZNF219 |
| RPL8 | TWSG1 |
| C8orf82 | ACTRT3 |
| ETS2 | HEY2 |
| P4HA2 | GPC6 |
| DAB2IP | GPR176 |
| MCUR1 | PDPN |
| PLOD3 | SPRED1 |
| PHLDB1 | CCND1 |
| PCBP3 | ZRANB3 |
| ZNF652 | LFNG |
| ZFTRAF1 | PLXDC2 |
| PPP1CA | PRDM6 |
| SLC30A1 | CKS1B |
| CRY2 | CAV1 |
| MPND | ENOX1 |
| BNIP3L | IRF8 |
| MLX | BAG2 |
| ATP5F1D | MARCKS |
| NBEAL2 | DCBLD2 |
| TMEM150A | SETBP1 |
| TNFRSF1A | GRASLND |
| LONP1 | FLVCR1-DT |
| HOXB4 | CCND2 |
| ADRA2C | NRG2 |
| RECQL5 | HOXD11 |
| GUCY1B1 | BAMBI |
| PPP1R13L | TCF7 |
| FRMD8 | TIMP2 |
| MTCH1 | CAMK2N1 |
| ARMC6 | PRDM1 |
| C9 | GOLIM4 |
| DUS1L | TTYH3 |
| GAREM1 | ADAMTS3 |
| EML3 | FBXO5 |
| STK40 | RAB31 |
| CATSPERG | PCDH19 |
| TBCB | PDE3A |
| LIN37 | FAT1 |
| HDAC11 | ANTXR1 |
| USP9X | PTOV1-AS1 |
| TMEM134 | TMEM120B |
| TRIOBP | GGH |
| MXRA7 |  |
| PSENEN | MAFB |
| FRS3 | ACP5 |
| TSC22D4 | CPQ |
| TBX2 | TACC3 |
| DESI1 | ATP2B1 |
| ACO2 | TBX3 |
| ARSD | PRRX1 |
| HGS | PCNA |
| DNAJB2 | BCL11B |
| SP2-DT | ZGRF1 |
| ACD | LINC01270 |
| TMEM222 | FCMR |
| SELENOW | FAM72D |
| MNT | SCAMP5 |
| INPP5A | HERC5 |
| INKA2 | MRGPRF |
| SOCS2-AS1 | KLRG1 |
| SNX21 | LINC01132 |
| TAF6L | CBX5 |
| USP47 | TENM3 |
| NUCB1 | CCN1 |
| ELL | MARCKSL1 |
| SLC12A2 | ANKRD50 |
| SMPD1 | EML6 |
| SPATA13 | H2BC3 |
| ANKRD39 | PGR |
| OGDH | PEG10 |
| WDR1 | ZNF724 |
| CHST3 | COL7A1 |
| RUVBL2 | ATRNL1 |
| RPN1 | H3C8 |
| TMEM205 | TIPIN |
| MED25 | LEF1 |
| YARS2 | EXTL3-AS1 |
| ULK3 | NRIP3 |
| ATP6V0D1 | FOXL2NB |
| DPH1 | SSX2IP |
| TRMT1 | RBM11 |
| SESN1 | MRGPRF-AS1 |
| SREBF2 | NID2 |
| SPATA9 | ARMH4 |
| SLC2A1-DT | CENPN |
| ITPR1 | TOX-DT |
| RPL10 | MTFR2 |
| C11orf54 | JAKMIP2 |
| CNTROB | NBL1 |
| PLCD1 | WDR62 |
| ARHGAP1 | ADGRV1 |
| TPRG1 | DNAJB5 |
| ZER1 | TGFB3 |
| DYNC2LI1 | RAB30 |
| FGGY |  |
| ENTPD6 | ARHGAP28 |
| RAB9A | ASPHD2 |
| H2BC21 | NPAS3 |
| DOCK9 | LRFN5 |
| THAP7 | FRY |
| CBX4 | H2BC7 |
| RNF227 | TUBB2B |
| KEAP1 | NXPH3 |
| TPK1 | KHK |
| PPM1B | FANCB |
| MAP2K7 | MMP16 |
| TNS2-AS1 | LBH |
| LPIN2 | SLFN13 |
| PMVK | DPP6 |
| TOM1L2 | PRR11 |
| ERICH2-DT | PLXDC1 |
| TIGD2 | NRCAM |
| GAPDH | SLC2A13 |
| FAM131A | HHIP |
| BRMS1 | ZFP69B |
| KAT2B | GCLC |
| SPACA9 | THBS1 |
| MAP3K14 | FAM72C |
| DDIT3 | FANCA |
| ATXN7L3 | ST6GAL2 |
| RPS6KB2 | SETBP1-DT |
| SFMBT2 | LINC02126 |
| HEBP2 | PGM2L1 |
| OSER1-DT | PTGES |
| MXD4 | PIK3R3 |
| WDR25 | LINC01391 |
| MUS81 | CDCA3 |
| KANK1 | SESN3 |
| ZMYND19 | SYT11 |
| HOXB2 | STC2 |
| TBC1D17 | IRS1 |
| GLCCI1 | MEX3B |
| CRELD2 | EGR2 |
| TNKS1BP1 | ACTN1-DT |
| PGLS | PIMREG |
| ARFRP1 | ZNF608 |
| CBX7 | GPC2 |
| CAMTA2 | DTL |
| BCR | BFSP1 |
| IRF2 | GSDMA |
| JAG1 | ADAMTS19-AS1 |
| HAND2-AS1 | RAI14 |
| TMBIM6 | PCSK5 |
| ANO10 | ADAMTS19 |
| CTSA | CNN1 |
| MSMO1 | BNC2 |
| EMP2 | BHLHE41 |
| SLC25A45 | AURKB |
| HOXB5 | NUSAP1 |
| PNPLA8 | PCDH10 |
| C1orf35 | CDC25C |
| ID2 | MLLT11 |
| NID1 | SNCA |
| DNAH2 | PHF21B |
| STK32C | CCNE2 |
| CHSY3 | BCL11A |
| SF3A2 | OSR1 |
| CDC42EP4 | ANLN |
| COX4I1 | BRCA2 |
| CHKA | KIF20A |
| ATP5MK | ZNF536 |
| FBXL8 | ZNF367 |
| SLC25A25 | CRTAC1 |
| DNAH9 | LMNB1 |
| TUT7 | HMOX1 |
| HADHB | H1-1 |
| DDX54 | CA11 |
| PIGS | CDC45 |
| MEF2D | MSC |
| SLF2 | CDCA2 |
| ABHD12 | ILDR2 |
| SYNGR1 | BMP7 |
| CDC42BPB | RRM2 |
| SNRNP70 | MSX2 |
| ACSS1 | EGR3 |
| PSMD8 |  |
| WDR83OS |  |
| CDC37 | CACNA1G-AS1 |
| DHPS | NRP2 |
| ATP6V0E1 | CRABP2 |
| RETREG2 | EPHA7 |
| ZNF276 | PKD2L1 |
| AGFG2 | MALT1-AS1 |
| PHKA2 | MFAP4 |
| CCDC159 | CDH2 |
| SLC16A1-AS1 | TENM4 |
| TMEM179B | PMEPA1 |
| DUSP3 | HOXC8 |
| F3 | ENC1 |
| CLBA1 | DMBX1 |
| MOB3A | GDNF |
| PARD6B | DENND5B-AS1 |
| SEPHS2 | SDK2 |
| ATP5F1E | COL8A1 |
| TOB2 | IGF2BP1 |
| CSNK1D | NKILA |
| AP2A1 | HS3ST3A1 |
| PIN1 | MEOX2 |
| SNHG7 | MCIDAS |
| SERTAD3 | FLRT1 |
| DNAJC27-AS1 | FAM222A |
| SULF2 | RPSAP52 |
| CTDSP2 |  |
| CFAP300 |  |
| MIB2 |  |
| FOXO3 |  |
| PDK2 |  |
| TMEM50A |  |
| MYL6 |  |
| ZNF783 |  |
| POLR2I |  |
| ALAD |  |
| SLC38A2 |  |
| DNM2 |  |
| CYBC1 |  |
| STOM |  |
| PPP1CB |  |
| ZNF775 |  |
| STON1 |  |
| GUCD1 |  |
| ESYT2 |  |
| TMCO6 |  |
| GMPPA |  |
| CACNB1 |  |
| RELA |  |
| CNOT11 |  |
| RCE1 |  |
| KCTD21 |  |
| ZCCHC2 |  |
| ALG12 |  |
| FAM98C |  |
| SH3BP5L |  |
| HOOK2 |  |
| NPC1 |  |
| SAMD4A |  |
| UBA52 |  |
| JMJD6 |  |
| TOLLIP |  |
| USP25 |  |
| BCL2L1 |  |
| RAD9A |  |
| TRAPPC1 |  |
| MTUS1 |  |
| ARPC3 |  |
| AKNA |  |
| ARFGAP2 |  |
| WASF2 |  |
| UBC |  |
| ARF5 |  |
| WSB2 |  |
| RGL3 |  |
| COL4A1 |  |
| ITPRID2 |  |
| NCOA2 |  |
| SRGAP2B |  |
| PCGF1 |  |
| HSD17B11 |  |
| RPL18A |  |
| OSR2 |  |
| ANAPC2 |  |
| TMEM165 |  |
| GPR155 |  |
| AKT1S1 |  |
| TRMT112 |  |
| HECTD3 |  |
| ATG4B |  |
| NFKBIA |  |
| MAPK14 |  |
| FUZ |  |
| CDK9 |  |
| INPPL1 |  |
| SLC19A2 |  |
| MKRN1 |  |
| PDCL |  |
| EIF1 |  |
| MATCAP2 |  |
| CAP1 |  |
| MEPCE |  |
| SYVN1 |  |
| MAEA |  |
| RETREG3 |  |
| IL10RB |  |
| SLC66A2 |  |
| HNRNPF |  |
| H1-0 |  |
| AP3D1 |  |
| TRMT10A |  |
| CASP9 |  |
| TGFBR2 |  |
| SUN1 |  |
| P4HB |  |
| TRIM8 |  |
| OTUB1 |  |
| LINC00472 |  |
| TMED5 |  |
| KMT5A |  |
| PSMB4 |  |
| BROX |  |
| PPP2R1A |  |
| BSDC1 |  |
| SLC35A4 |  |
| SNX25 |  |
| VPS13C |  |
| TYSND1 |  |
| ZNF768 |  |
| ZNF57 |  |
| ALKBH5 |  |
| MYL12B |  |
| ANKRD27 |  |
| ACLY |  |
| ZNHIT1 |  |
| CIPC |  |
| SERAC1 |  |
| NOP53 |  |
| PPP1R7 |  |
| SNRNP35 |  |
| WDR27 |  |
| ARHGAP21 |  |
| COX6B1 |  |
| FBRS |  |
| AMMECR1L |  |
| SNX7 |  |
| MEF2A |  |
| PAN2 |  |
| DAZAP2 |  |
| DCAKD |  |
| RREB1 |  |
| DIP2A |  |
| TPT1 |  |
| CFLAR |  |
| STX5 |  |
| CXXC5 |  |
| RMC1 |  |
| THBS3 |  |
| TRIM35 |  |
| CHST12 |  |
| HIF1A |  |
| TMEM9B |  |
| ZDHHC3 |  |
| LTO1 |  |
| SAP30BP |  |
| ATG13 |  |
| P2RX4 |  |
| PDPK1 |  |
| NRBF2 |  |
| MED9 |  |
| PEF1 |  |
| CEP68 |  |
| ZBTB11-AS1 |  |
| TBC1D22A |  |
| KDM3A |  |
| DYNLT1 |  |
| SRGAP2C |  |
| C1orf43 |  |
| ACADVL |  |
| FBXW7 |  |
| RC3H2 |  |
| RPL12 |  |
| ZNF263 |  |
| PPP1R3D |  |
| SMIM14 |  |
| HEXIM1 |  |
| ARL6IP6 |  |
| ATP5PD |  |
| FYN |  |
| UGP2 |  |
| OXSR1 |  |
| ARL2BP |  |
| BICD2 |  |
| DEDD |  |
| ZNF487 |  |
| FAM114A1 |  |
| MCL1 |  |
| TMEM181 |  |
| SRGAP2 |  |
| DHTKD1 |  |
| ARNT |  |
| AHCYL1 |  |
| UBE2H |  |
| CALCOCO2 |  |
| WASHC4 |  |
| COPS7A |  |
| MAVS |  |
| GLUD1 |  |
| FHIP1B |  |
| STK35 |  |
| C6orf120 |  |
| MRTFA |  |
| H3-3B |  |
| YWHAZ |  |
| DDAH1 |  |
| ZNF672 |  |
| TMOD3 |  |
| RMND5A |  |
| DCUN1D2 |  |
| DNAJC1 |  |
| CD27-AS1 |  |
| TSHZ1 |  |
| LYSMD1 |  |
| HDLBP |  |
| GLYR1 |  |
| EXOSC7 |  |
| CIRBP |  |
| PPP1R15B |  |
| TMEM59 |  |
| PRKAR2A |  |
| TXNDC11 |  |
| ZFAND5 |  |
| NR2C1 |  |
| MYO9A |  |
| ZZEF1 |  |
| SAFB |  |
| MAP3K12 |  |
| MTMR3 |  |
| OGA |  |
| ZNF394 |  |
| NRAS |  |
| RBM39 |  |
| RALA |  |
| RAB5B |  |
| FARP2 |  |
| CUL4A |  |
| ADD1 |  |
| CD46 |  |
| WDR82 |  |
| VPS4B |  |
| HGSNAT |  |
| PDCD6IP |  |
| CFDP1 |  |
| CDS2 |  |
| EMC2 |  |
| ITSN1 |  |
| POLR1D |  |
| ABI1 |  |
| CRCP |  |
| RSPH3 |  |
| UBR4 |  |
| SETD3 |  |
| NUP85 |  |
| DNAAF9 |  |
| CCNL1 |  |
| WASHC2A |  |
| EIF2D |  |
| DICER1 |  |
| MMS19 |  |
| EIF4E2 |  |

## AR is primarily located in enhancer regions in the genome of PCOS stromal cells


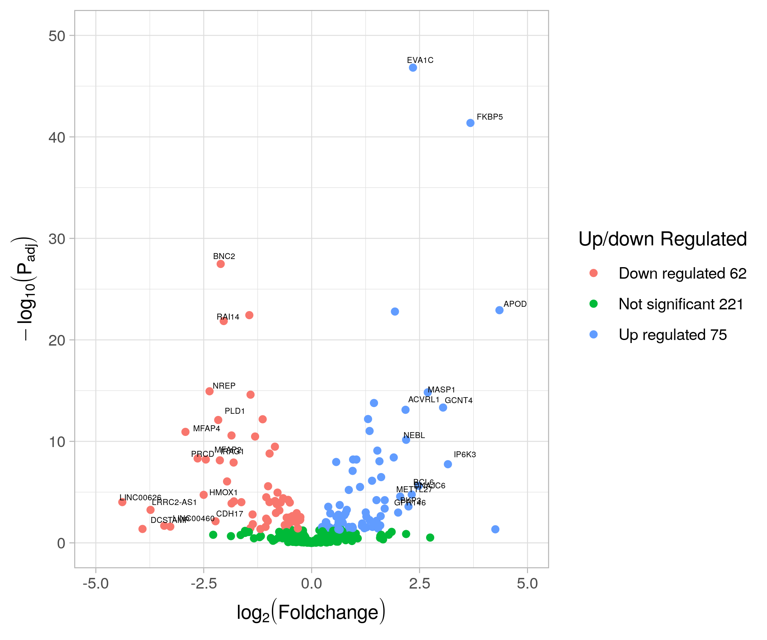


Figure S1 Volcano plot showing differentially expressed genes with AR peaks in promoter region

*WT1 and AR binding sites are collocated*
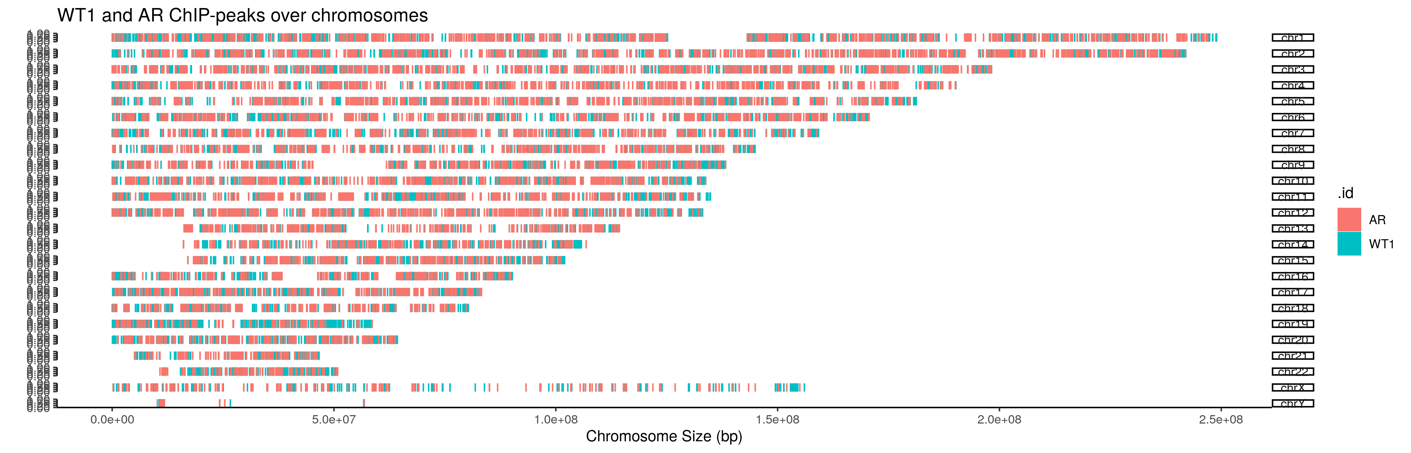


Figure S2 Genome wide location of WT1 and AR peaks


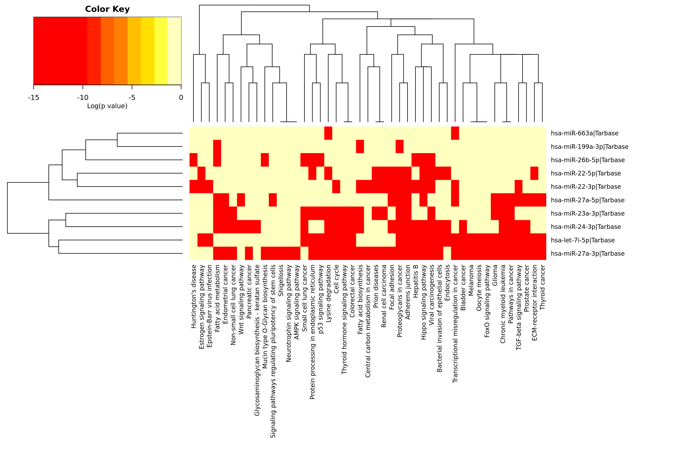


Figure S3 . Enriched pathways for miRNAs with WT1 peaks in promoters (from DIANA-miRPath database with the KEGG gene set


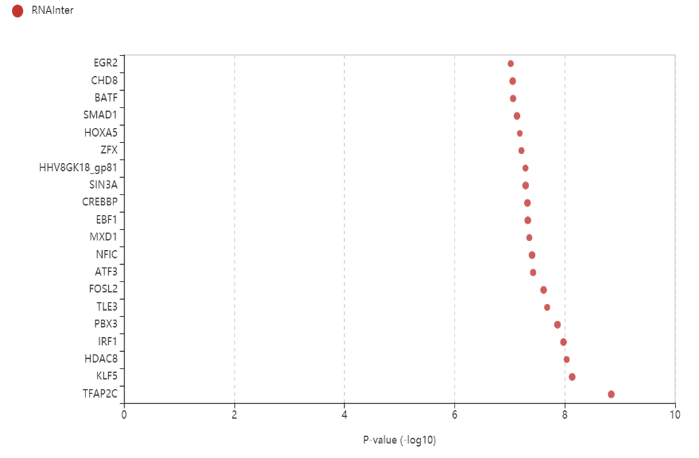


Figure S4 Genes associated by lncRNAs with WT1 peaks in promoters (from LncSEA database).

Table S3 Genes with overlapping WT1/AR peak in promoter region

| **Genes with overlapping WT1/AR peak in promoter regions** |
| --- |
| ACTN1-AS1 |
| ACVRL1 |
| AMOTL2 |
| APTX |
| BAHCC1 |
| BCL6 |
| C1RL |
| C1RL-AS1 |
| CCAR2 |
| CD81-AS1 |
| CDC40 |
| CLECL1 |
| COL7A1 |
| CREB1 |
| CROCC |
| CTBP1-AS |
| DARS1 |
| DDX11L1 |
| DENND6A |
| DENND6A-DT |
| DNAJA1 |
| DPP3 |
| DUSP1 |
| E2F6 |
| EIF2D |
| EOGT |
| FAM86EP |
| FANCA |
| FAP |
| FBLIM1 |
| FKBP5 |
| GADD45B |
| GPR146 |
| HMOX1 |
| HS1BP3-IT1 |
| IL1R1 |
| IRF2BP2 |
| ITGA5 |
| ITPKC |
| JSRP1 |
| LAMP1 |
| LINC00886 |
| LINC01117 |
| LINC01132 |
| LINC01714 |
| LINC01800 |
| LINC02026 |
| LINC02126 |
| LINC02139 |
| LINC02259 |
| LINC02453 |
| LYPD3 |
| METTL27 |
| MFAP4 |
| MIR199A1 |
| MIR22 |
| MIR26B |
| MIR3648-1 |
| MIR3935 |
| MIR4289 |
| MIR4530 |
| MIR548AL |
| MIR663A |
| MIR6724-1 |
| MIRLET7I |
| NBPF10 |
| NBPF19 |
| NCOR2 |
| NEAT1 |
| NFKBIA |
| NOTCH2NLA |
| NOTCH2NLC |
| OSMR |
| OSMR-AS1 |
| PGM2L1 |
| PHLDB1 |
| PKNOX2-AS1 |
| PKP3 |
| PLEKHG2 |
| RNU2-1 |
| RPL37 |
| S100A2 |
| SCGB1A1 |
| SIPA1 |
| SMARCA2 |
| SNORD3D |
| SNORD72 |
| SPIRE2 |
| SPON2 |
| STAT3 |
| SYNC |
| SYS1 |
| TBX3 |
| THBS1 |
| TPRG1 |
| TPRG1-AS1 |
| UBC |
| WASF1 |
| WWTR1-AS1 |
| ZFYVE16 |

Table S4 miRNAs and lncRNAs with WT1/AR in promoter regions

| **miRNA** | **lncRNA** |
| --- | --- |
| MIR199A1 | LINC00886 |
| MIR22 | LINC01117 |
| MIR23A | LINC01132 |
| MIR24-2 | LINC01714 |
| MIR26B | LINC01800 |
| MIR27A | LINC02026 |
| MIR3648-1 | LINC02126 |
| MIR3935 | LINC02139 |
| MIR4289 | LINC02259 |
| MIR4505 | LINC02453 |
| MIR4530 |  |
| MIR548AL |  |
| MIR663A |  |
| MIR6724-1 |  |
| MIRLET7I |  |

## Co-localisation of WT1 and AR corresponds with H3K4me3 and H3K27ac histone modifications in cis-regulatory elements

Table S5 Number of WT1 and WT1/AR peaks overlapping H3K4me3 and H3K27ac histone mark peaks in hESC treated with cAMP and MPA

|  | **Number of WT1 Peaks** | **Number of WT1/AR Peaks** |
| --- | --- | --- |
| **Inside of promoter regions** | 6,676 | 106 |
| **Outside of promoter regions** | 12,741 | 720 |
| **Inside of promoter regions Overlapping H3K4me3 enriched regions** | 5,180 | 59 |
| **Outside of promoter regions Overlapping H3K27ac enriched regions** | 2,054 | 165 |

**Methods**

## Analysis of ChIP-seq data

| 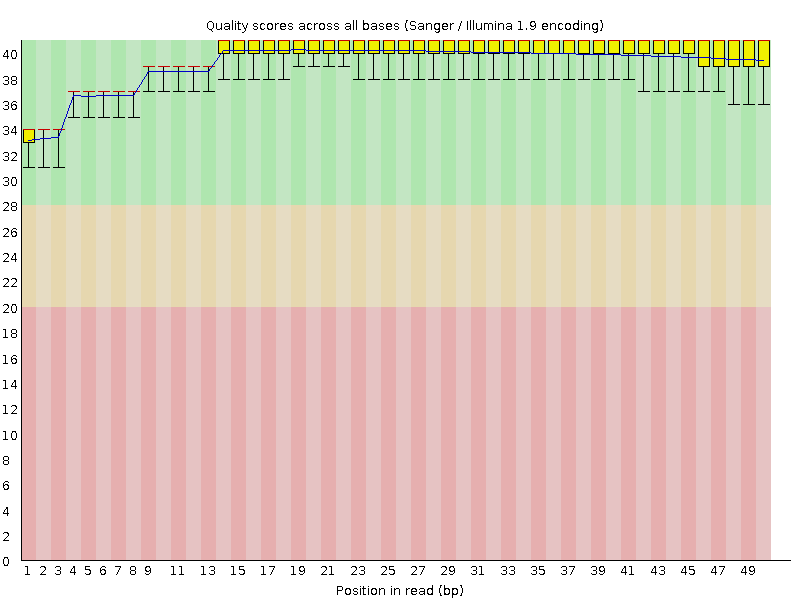  A | 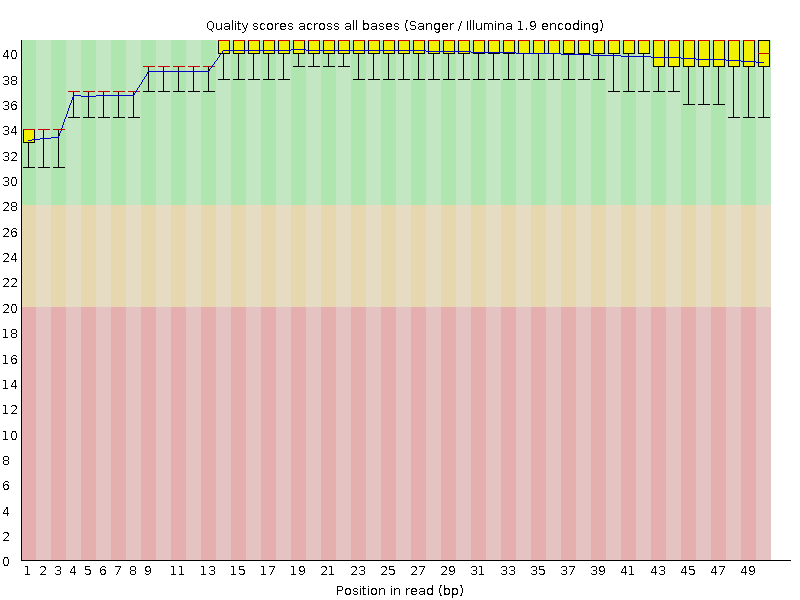  B | 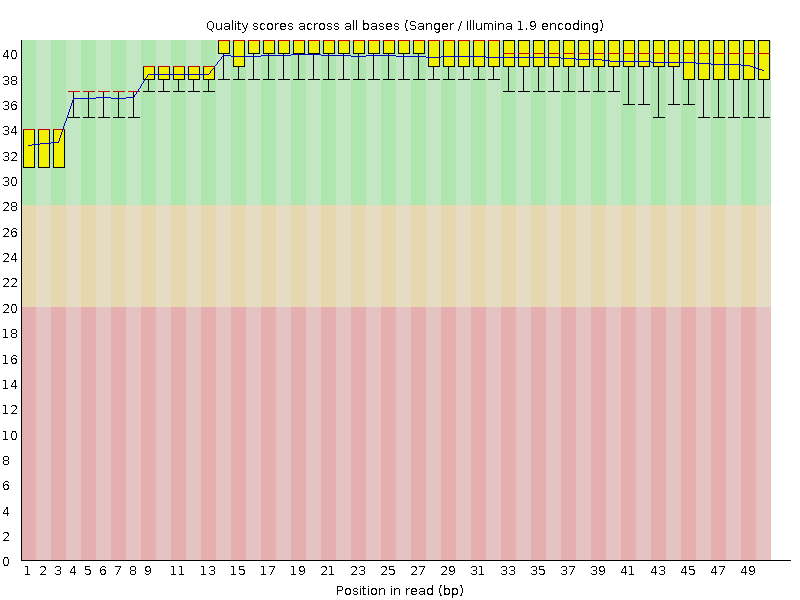  C |
| --- | --- | --- |

Figure 5 FASTQC per base quality for A) WT1 hESC ChIP-seq sample, B), AR hESC ChIP-seq sample C) hESC input sample

| 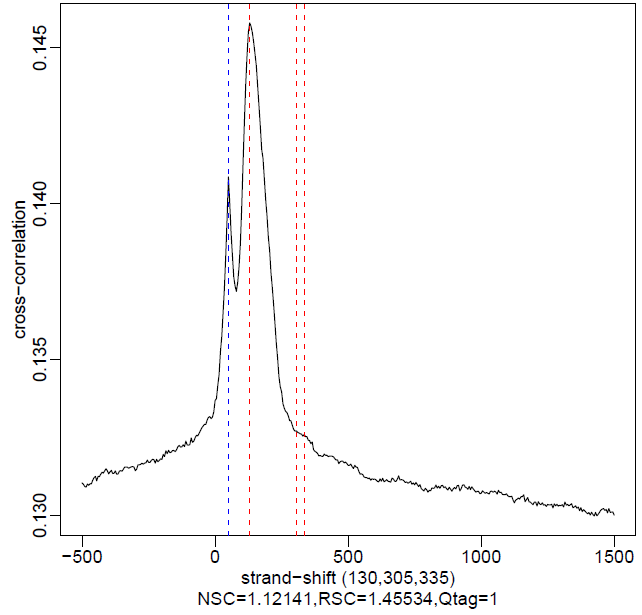  A | 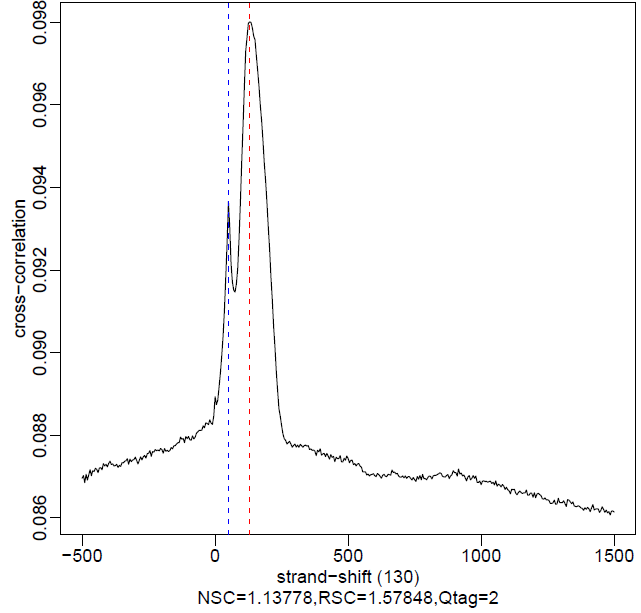  B | 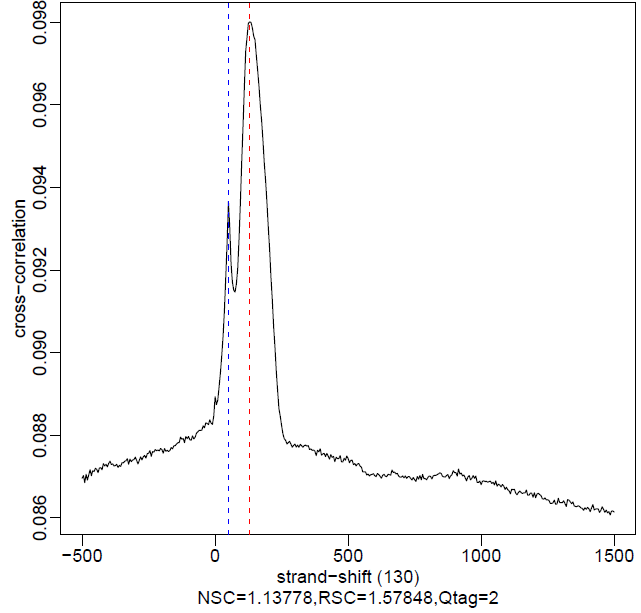  C |
| --- | --- | --- |

Figure 6 Phantom peak quant tools correlation analysis results for A) WT1 hESC ChIP-seq sample, B), AR hESC ChIP-seq sample C) hESC input sample
